# Socio-Ecological Factors Associated with Dengue Risk and *Aedes aegypti* Presence in the Galápagos Islands, Ecuador

**DOI:** 10.3390/ijerph16050682

**Published:** 2019-02-26

**Authors:** Sadie J. Ryan, Catherine A. Lippi, Ryan Nightingale, Gabriela Hamerlinck, Mercy J. Borbor-Cordova, Marilyn Cruz B, Fernando Ortega, Renato Leon, Egan Waggoner, Anna M. Stewart-Ibarra

**Affiliations:** 1 Quantitative Disease Ecology and Conservation (QDEC) Lab, Department of Geography and Emerging Pathogens Institute, University of Florida, Gainesville, FL 32601, USA; clippi@ufl.edu (C.A.L.); ghamerlinck@ufl.edu (G.H.); 2 Institute for Global Health and Translational Sciences, SUNY Upstate Medical University, Syracuse, NY 13210, USA; nightinr@upstate.edu (R.N.); egan.waggoner@cadmusgroup.com (E.W.); stewarta@upstate.edu (A.M.S.-I.); 3 Escuela Superior Politecnica del Litoral (ESPOL), Facultad de Ingenieria Maritima y Ciencias del Mar, Guayaquil 090150, Ecuador; meborbor@espol.edu.ec; 4 La Agencia de Regulación y Control de la Bioseguridad y Cuarentena para Galápagos (ABG), Puerto Ayora, Galápagos 200350, Ecuador; marilyn.cruz@abgalapagos.gob.ec; 5 School of Public Health, Universidad San Francisco de Quito, Quito 170901, Ecuador; fortega@usfq.edu.ec; 6 Laboratorio de Entomología Médica & Medicina Tropical, LEMMT, Universidad San Francisco de Quito, Quito 170901, Ecuador; rleon@usfq.edu.ec

**Keywords:** dengue fever, *Aedes aegypti*, social-ecological risk, islands, Galápagos, Ecuador

## Abstract

Dengue fever is an emerging infectious disease in the Galápagos Islands of Ecuador, with the first cases reported in 2002 and subsequent periodic outbreaks. We report results of a 2014 pilot study conducted in Puerto Ayora (PA) on Santa Cruz Island, and Puerto Baquerizo Moreno (PB) on San Cristobal Island. To assess the socio-ecological risk factors associated with dengue and mosquito vector presence at the household level, we conducted 100 household surveys (50 on each island) in neighborhoods with prior reported dengue cases. Adult mosquitoes were collected inside and outside the home, larval indices were determined through container surveys, and heads of households were interviewed to determine demographics, self-reported prior dengue infections, housing conditions, and knowledge, attitudes, and practices regarding dengue. Multi-model selection methods were used to derive best-fit generalized linear regression models of prior dengue infection, and *Aedes aegypti* presence. We found that 24% of PB and 14% of PA respondents self-reported a prior dengue infection, and more PB homes than PA homes had *Ae. aegypti*. The top-ranked model for prior dengue infection included several factors related to human movement, household demographics, access to water quality issues, and dengue awareness. The top-ranked model for *Ae. aegypti* presence included housing conditions, mosquito control practices, and dengue risk perception. This is the first study of dengue risk and *Ae. aegypti* presence in the Galápagos Islands.

## 1. Introduction

Dengue fever is a mosquito-borne viral illness that causes an estimated 96 million new apparent (symptomatic) infections per year worldwide, with 16 million infections in the Americas annually [1,2]. Dengue increased in geographic distribution and incidence in recent decades [3]. Dengue infections typically present with febrile symptoms, and, although rare, severe cases of dengue can be fatal, resulting in an estimated 20,000 deaths per year worldwide [4]. Recently, Bhatt and colleagues [1] estimated an additional 294 million inapparent cases occurred in 2010, where individuals either experienced mild symptoms or were asymptomatic. These individuals would not have been detected by regular public health surveillance and, therefore, represent a large potential infection reservoir. The impact of these individuals is important to consider when assessing social and ecological (socio-ecological) factors associated with dengue risk and mosquito vector presence. 

The dengue virus (DENV, family *Flaviviridae*, genus *Flavivirus*) is primarily transmitted by *Aedes* spp. mosquitoes. In recent years, new arboviruses transmitted by the same mosquito vectors (*Ae. aegypti* and *Ae. albopictus*) emerged in the Americas, including the chikungunya (CHIKV) and Zika (ZIKV) viruses [5]. Mosquito control by the public health sector is the primary means of controlling arbovirus outbreaks in the Americas. Control programs maintained by local health/government offices and organizations typically include fumigation to control adult mosquitoes, larvicide applications, and active mosquito surveillance. Unfortunately, existing vector control efforts are largely unsuccessful at preventing epidemics. Furthermore, abatement programs that rely heavily on chemical control methods are also problematic due to the rise of insecticide resistance in *Aedes* populations [6]. Emerging insecticide resistance can further drive intervention failure while wasting public health agency resources [7]. New strategies that consider the nuances of socio-ecological conditions and risk factors are urgently needed to inform local targeted control campaigns in the context of household-level strategies and behaviors. 

In Ecuador, dengue is transmitted primarily by the *Ae. aegypti* mosquito vector, an urbanized anthropophilic mosquito. The dengue virus and *Ae. aegypti* were eradicated on mainland Ecuador in the 1950s through successful dichlorodiphenyltrichloroethane (DDT) campaigns [8]. Following drastic reductions in vector control programs and rapid, uncontrolled urbanization in the 1970s and 1980s, dengue re-emerged in Ecuador in 1989; by the early 2000s, all four dengue virus serotypes co-circulated in the coastal lowland mainland region [9,10,11]. The primary means of preventing dengue transmission in Ecuador is through vector control, by reducing the density of *Ae. aegypti* in high-risk households, since a dengue vaccine is not yet available for widespread use. Dengue control is conducted by the Ministry of Health, using repeated cycles of ultra-low-volume fumigation of neighborhoods throughout the rainy (peak transmission) season, and indoor residual spraying in and around homes with suspected dengue cases. The Ministry of Health also conducts routine visits to homes to apply larvicide (temefos/abate) to water-bearing containers and destroys larval habitat in and around homes. 

Dengue emerged on the Galápagos Islands of Ecuador in the early 2000s [12]. Islands are valuable epidemiological study locations as they can be used to study outbreaks of disease in relative isolation. This approach also allows for the identification of socio-ecological factors associated with disease transmission. Dengue was also found on the relatively isolated Easter Island of Chile and the Hawaiian Islands of the United States. These islands are ideal case studies for measuring the response of disease transmission to local factors without regional influences [13].

**Figure 1:**
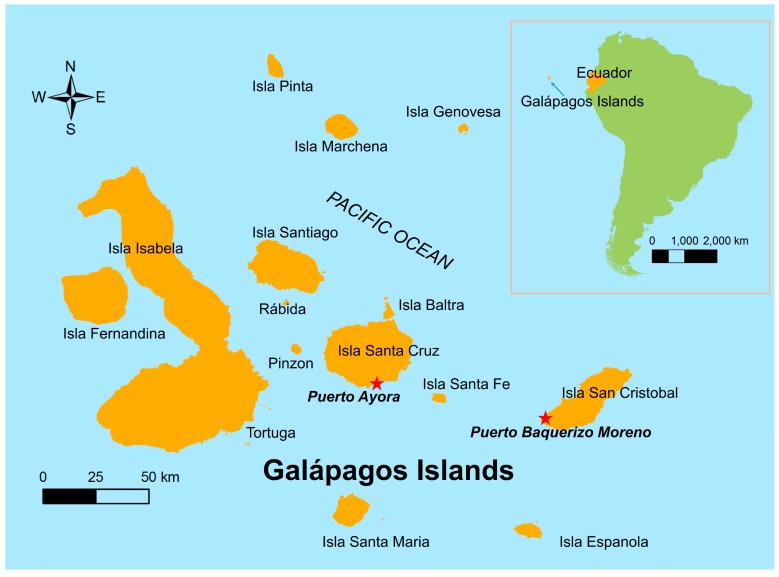
Location of study sites (Puerto Ayora and Puerto Baquerizo, red stars) in the Galápagos Islands, Ecuador.

The Galápagos Islands of Ecuador, a World Heritage Site, are located 1000 kilometers from the mainland (Figure 1). The islands are renowned for their biodiversity due to their relative isolation and minimal impact by people, who first settled on the islands in the mid-1800s [14]. In recent decades, the population increased rapidly, from 1346 people in 1950 [14] to 30,890 people residing on four islands in 2017 [15]. Tourism is the most important economic activity [14], with 215,691 tourists visiting the islands in 2014 [16]. Invasive species introduced to the islands through human activity are recognized as a serious threat to endemic species on the islands [17]. The introduction of pathogens is an additional concern, but previous work focused primarily on pathogen impact on native animals (e.g., West Nile virus risk to an endemic bird species [18,19,20]). Less attention is paid to emerging pathogens in humans.

The central vector control team for the Galápagos is based in Puerto Ayora, due to the greater burden of disease, and a smaller team is based in Puerto Baquerizo Moreno. Household-level *Ae. aegypti* control interventions require significant investment of resources including financial resources, personnel, field transportation, chemicals, and material supplies. The Ministry of Health in the Galápagos engages in multisectoral collaborations for dengue prevention, including dengue education in local schools in partnership with the Ministry of Education, and community clean-up campaigns with private institutions and non-profit organizations.

The aim of our study was to identify socio-ecological factors that were associated with an increased risk of dengue fever transmission and *Ae. aegypti* presence in households of Puerto Ayora and Puerto Baquerizo Moreno of the Galápagos Islands. To our knowledge, this is the first report on dengue fever risk in the Galápagos Islands. Prior studies in other Latin American locations showed that risk factors such as water storage practices, knowledge and risk perception, human movement patterns, and housing conditions influence vector abundance and risk of dengue infection [21,22,23,24,25]. Identifying household-level risk factors for dengue transmission in the Galápagos would, therefore, provide specific targets for public health interventions on the islands.

## 2. Materials and Methods 

### 2.1. Study Site

**Figure 2:**
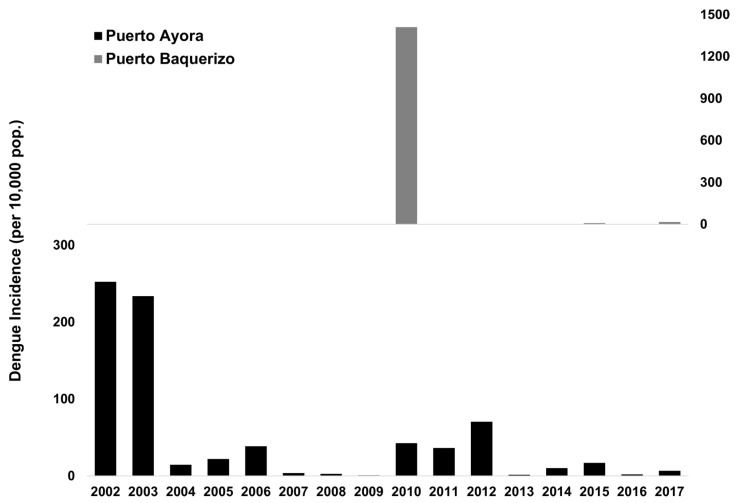
Annual incidence of dengue infections in the Galápagos Islands, showing Puerto Ayora on Santa Cruz Island, and Puerto Baquerizo on San Cristobal Island (data provided by the Ministry of Health).

Only three mosquito species were reported to be present on the Galápagos islands: *Aedes aegypti*, *Ae. Taeniorhynchus*, and *Culex quinquefasciatus* [26,27]. The *Ae. aegypti* mosquito is believed to have been introduced to Santa Cruz Island in the 1990s [27]. The first outbreak of dengue in the Galápagos Islands occurred in 2002 in the city of Puerto Ayora on Santa Cruz Island, the most populated city center in the Galápagos (Figure 1 and Figure 2). A total of 227 cases were reported during the outbreak, resulting in an incidence rate of 252 cases per 10,000 people. There were cases of dengue reported from Puerto Ayora every year since 2002, as well as periodic outbreaks (Figure 2). Dengue cases are clinically diagnosed, and suspected cases trigger an epidemiological investigation and vector control. Arbovirus case data was provided by the Ministry of Health. From 2003 to epidemic week (EW) 33 of 2018 a total of 540 cases have been reported, resulting in an average incidence rate of 40 cases per 10,000 people [28]. A proportion of cases are thought to be imported from the mainland where dengue is hyper-endemic; however, *Ae. aegypti* is well established on the islands and the periodic outbreaks suggest locally acquired infections. 

Puerto Ayora is considered to be within the dry lowland climatic zone [29]. Dengue transmission in Puerto Ayora is highly seasonal. Cases peak in June, following the hot season from February to May (23 °C to 30 °C, mean monthly rainfall = 63 mm; 2002–2014, Figure 3). 

Chikungunya and Zika viruses emerged for the first time on Santa Cruz in 2015 and 2016, respectively. There were 25 cases of chikungunya and two cases of Zika. Of the 25 chikungunya cases, 20 were confirmed by PCR at the national reference laboratory (INSPI) in Guayaquil, and five were confirmed by clinical presentation and epidemiological nexus. All Zika cases were laboratory-confirmed by PCR at the national reference laboratory (INSPI) in Guayaquil.

San Cristobal, the second most populated Galápagos island (Figure 1), experienced a major dengue outbreak in 2010 in the capital city of Puerto Baquerizo Moreno. A total of 941 cases were reported, resulting in an incidence rate of 1410 cases per 10,000 people [28]. From 2014 to 2018, 25 cases of dengue fever were reported (Figure 2). Since only one large outbreak was reported, the seasonality of transmission is unknown, although climate conditions are similar to Puerto Ayora. In 2017, three cases of Zika virus were reported. No cases of dengue, Zika, or chikungunya were reported from the other inhabited islands of Floreana and Isabela. 

In August and September 2014, we surveyed 100 households on two of the Galápagos Islands. Fifty households in Puerto Ayora (PA) (latitude: −0.7402, longitude: −90.31, elevation: 15 m) and 50 households in Puerto Baquerizo (PB) (latitude: −0.9232, longitude: −89.60, elevation: 6 m) were included. PA and PB are the largest population centers in the Galápagos Islands (2010 population estimates: 11,974 and 6672, respectively [30]), and the only sites in the Galápagos where cases of autochthonous dengue were reported. Surveyed households were in a sector of each city that had historically high *Ae. aegypti* indices according to the local Ministry of Health, and homes were located approximately 200–250 meters apart, the flight range of the *Ae. aegypti* mosquito. 

### 2.2. Ethics Statement

The study protocol was reviewed and approved as exempt by the Institutional Review Board (IRB) of the SUNY Upstate Medical University, and approved by Universidad San Francisco de Quito (AP# 2014-061_IN). The protocol was also approved by the Agencia de Regulación y Control de la Bioseguridad y Cuarentena para Galapagos (ABG) prior to study start. Heads of households (>18 years of age) signed an informed consent form prior to study start.

### 2.3. Field Data Collection

Technicians surveyed heads of households to identify self-reported prior dengue infections in household members, demographics, risk perceptions, dengue knowledge, sources of dengue information, and household vector control practices. Housing conditions were also assessed and recorded by survey technicians. The survey instrument was developed from an instrument that was field tested in other cities in Ecuador [22], and was piloted with Ministry of Health technicians prior to study start (survey instruments in Spanish and English are available upon request).

We collected adult mosquitoes at each location and identified species using a stereo microscope. All adult mosquitoes were collected from inside and outside the house using prokopacks, highly effective lightweight backpack aspirators [31]. To assess the presence of immature mosquito life stages, we conducted standard container surveys to identify the prevalence of water-bearing containers with *Ae. aegypti* pupae and larvae in and around the home [22,32,33,34]. We recorded descriptive information about each container, including type, use, source of water, and location inside or outside the home. All pupae and a subset of larvae were reared to adulthood in the laboratory to confirm species identification. 

### 2.4. Statistical Models

Survey data were used to identify socio-ecological variables associated with the occurrence of (1) self-reported dengue fever (model: *dengue*) and (2) vector presence (model: *mosquito presence*); this is a combined measure of presence of either adult or juvenile *Aedes aegypti* mosquitoes. We hypothesized that both self-reported dengue and mosquito presence were associated with one or more of these factors (see Table 1). Survey responses were coded and grouped into five suites of variables: human movement, demographics, mosquito abatement practices, housing characteristics, and knowledge and attitudes. We used descriptive statistics to compare responses between the two sites, PA and PB, including Student’s independent *t*-test, Pearson’s chi-square test with Yates continuity correction, and Fisher’s exact test. All statistics were conducted in R 3.5.2 [35]. 

We used an information theoretical approach to derive best-fit models comprising explanatory variables for (1) self-reported dengue (model: *dengue*) and (2) presence of *Ae. aegypti* (juveniles and/or adults) at households (model: *mosquito presence*). To find the best-fit model for self-reported dengue infections, we used multi-model selection to determine which survey outcomes related to the five suites of variables (human movement, demographics, housing characteristics, mosquito abatement practices, and knowledge and attitudes) were associated with the number of self-reported dengue cases on both islands (Table 1). As the survey provided data only on current mosquito abatement practices, we were unable to use this suite of variables in the *dengue* model search. We used a similar approach to find the best-fit model for *Ae. aegypti* presence. We examined which survey factors related to housing conditions, knowledge and attitudes, and mosquito abatement practices were influencing the presence of *Ae. aegypti* within the homes of survey participants (Table 1). The variables from the human Movement and demographics suites were not used in the *mosquito presence* model search. All model selection processes were conducted using “glmulti”, an R package for multi-model selection [36], specifying a logistic modeling distribution in a generalized linear model (GLM) framework (GLM, family = binomial, link = logit). 

We excluded some factors from model variable candidates due to missing or uninformative data (i.e., if the responses were identical across all households). Model selection was run to convergence using glmulti’s genetic algorithm (GA); models were ranked using Akaike’s information criterion (AIC) corrected for small sample size (AICc). For each set of variables in our hypotheses, we obtained a best-fit model, along with multiple competing top models, using the threshold criteria of ΔAICc ≤ 2 (competing top models given in Tables S1 and S2, Supplementary Materials). We calculated parameter estimates, odds ratios (OR), and 95% confidence intervals (CI) for variables in the top-ranked model from each search. Variance inflation factors (VIF) and condition numbers (κ) were calculated for each top model to assess multicollinearity and model stability, respectively, with VIF values below 10 indicating low multicollinearity and condition numbers below 30 indicating model stability. 

## 3. Results

We interviewed the head of house of 100 households, where PA households (*n* = 50) represented 196 household members, and PB households (*n* = 50) represented 194 household members. At the household level, prior dengue infections were reported by more households in PB (28%) than in PA (20%), although the difference was not significant (*p* > 0.05, chi-square test). Most people on both islands reported seeking medical care when ill with suspected dengue (PA = 64%, PB = 78%, *p* > 0.05, Fisher’s exact test). 

Water-bearing containers (*n* = 248) were inspected for the presence of *Ae. aegypti* (PA = 119, PB = 129). Significantly more houses were found to have containers with juvenile *Ae. aegypti* in PB than in PA (*p* = 0.012, Table 2). The house index (number of homes with juvenile *Ae. aegypti* per 100 homes) was 20 in PB and 6 in PA (Table 2). The Breteau index (number of containers with juvenile *Ae. aegypti* per 100 homes) was 26 in PB and 6 in PA (Table 2). A greater proportion of surveyed containers were found with *Ae. aegypti* juveniles in PB than in PA (*p* = 0.019). The predominant characteristics of containers positive for juvenile *Ae. aegypti* (*n* = 16) were low water tanks made of cement or plastic (92%), containers that were completely or partially uncovered (92%), containers located outdoors (85%), containers that were shaded (85%), containers filled with tap water as opposed to rain water (100%), and containers intended for domestic use (i.e., used for cooking, cleaning, and laundry, as opposed to abandoned containers) (77%). Very few houses on either island had adult *Ae. aegypti* present (PA = 2, PB = 7). 

### 3.1. Risk Perceptions and Practices

Most households in PA (82%) and PB (94%) reported that dengue was a serious problem in their community (*p* = 0.1) and a severe disease (PA = 86%, PB = 98%, *p* = 0.06) (Table 1). Significantly more PB households reported that it was difficult or impossible to prevent dengue (*p* = 0.02, Table 1). The majority of heads of households knew that dengue was transmitted by a mosquito (94% on both islands), and most people had received information about dengue prevention from multiple sources. However, few people had participated in dengue prevention campaigns (PA = 12%, PB = 10%). Sources of dengue information were similar between islands, with media (television, newspaper, radio) as the primary source of information on both islands, and social networks as the least likely source of information (Table 1). 

Overall, PB households implemented more prevention strategies than PA households (PA mean = 1.48, SD = 0.76, PB mean = 3.76, SD = 2.09, *p* < 0.001). PB households reported several prevention strategies significantly more frequently than PA households, including use of screens on windows and doors (*p* < 0.001), topical repellent (*p* = 0.03), keeping the property clear of trash (*p* = 0.02), closing windows and doors (*p* < 0.001), cutting grass and plants (*p* = 0.01), adding chemicals to standing water (*p* = 0.004), and eliminating standing water (*p* < 0.001) (Table 1). Fumigation and use of mosquito nets were the least commonly reported mosquito control actions in both locations.

### 3.2. Model Selection Outcomes

**Figure 4:**
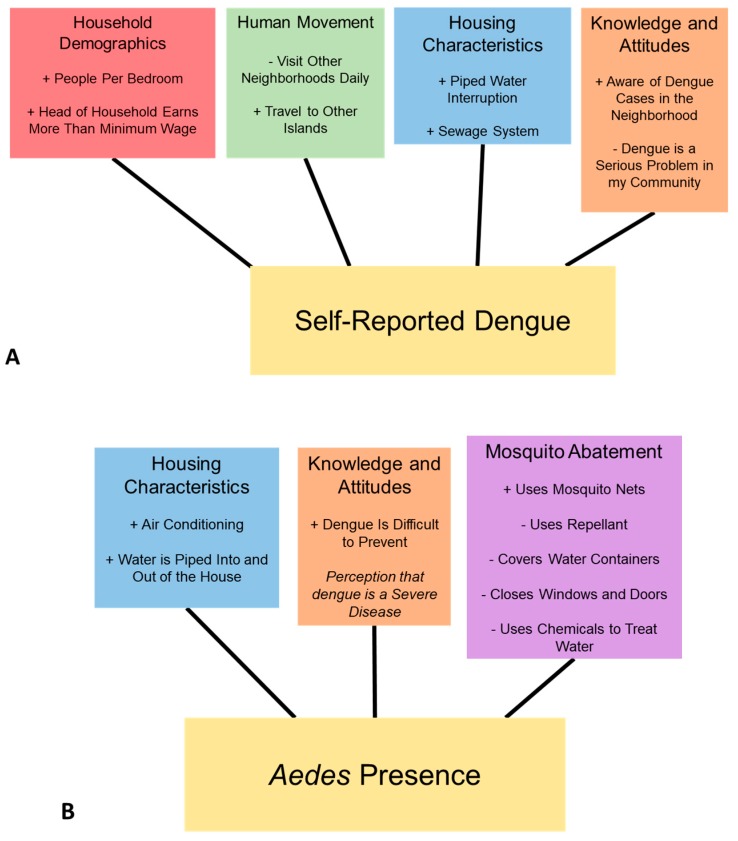
Suites of socio-ecological variables in the top selected models for (**A**) self-reported dengue cases, and (**B**) presence of *Aedes aegypti*; positive associations in the model are denoted (+), and negative associations (-); italics denotes an important factor but direction of association is not discernable.

The top-ranked model of a prior self-reported case of dengue in the household (AICc = 70.04, κ = 12.52) included the following suite of positively associated variables: the number of people per room in the home, the head of the household earning more than minimum wage, household members who travel between islands, frequent interruptions in the piped water supply, waste water disposal by a sewage system, and being aware of dengue cases in their community (Table 3, Figure 4). Having the perception that dengue is a serious problem in the community, and visiting other neighborhoods daily were negatively associated with self-reported dengue. Fourteen additional models were found within 2 AICc units of the top model (Supplementary Table S1). 

The top-ranked model to predict the presence of *Ae. aegypti* in and around households (AICc = 60.14, κ = 8.68) included the following positively associated variables: use of mosquito nets in the home, air conditioning, water being piped into and out of the house, and the perception that dengue is a severe and difficult disease to prevent, although this last parameter estimate was unreliable in the model (Table 4, Figure 4). Negatively associated variables included covering water containers, using repellants, closing doors and windows, and treating water with chemicals. Eight additional models were found within 2 AICc units of the top model, comprising alternating selections of similar variables to the top model (Supplementary Table S2).

## 4. Discussion

This study provides the first insights into the nature of household-level dengue fever risk and *Aedes aegypti* presence on the Galápagos Islands of Ecuador, where the disease emerged in the last 20 years. We found several suites of socio-ecological variables that Galápagos residents identified as important factors associated with reported dengue and were associated with mosquito presence (Figure 4): human movement, demographics, housing characteristics, knowledge and attitudes, and mosquito abatement practices. These findings can be readily interpreted and used to inform the design and implementation of targeted vector control campaigns that reflect the local social-ecological context [22,37].

We found that higher income and education of the head of the household were positively associated with frequent travel by household members and greater awareness of dengue. The role of human movement in dengue transmission was documented in prior studies in Iquitos, Peru [23,38]. We also found that self-reported prior dengue infections were associated with housing conditions, dengue awareness, and frequent travel by household members (Figure 4, Table 3). Increased general dengue awareness by residents may also inflate self-reported infections. However, we were able to identify many socio-ecological factors that are associated with self-reported dengue infections, as well as *Ae. aegypti* presence, which can offer valuable insights for prevention and control. 

Greater housing density (people per bedroom) and frequent interruptions in the piped water supply were also indicative of greater dengue risk. These variables likely reflect greater risk of exposure to infectious mosquito bites, due to larval habitat in water storage containers, and increased probability of infectious bites due to human crowding. These findings are consistent with other studies, both on mainland Ecuador [21,22,39] and elsewhere [40,41,42].

We found several important variables when assessing our model of *Ae. aegypti* presence, including housing conditions, dengue risk perception, and prevention practices. We found that homes were more likely to have *Ae. aegypti* if they had no screens on windows or doors, if they did not cover water containers, and if they perceived that dengue was difficult to prevent (Figure 4). These risk factors are consistent with prior studies from Ecuador, Taiwan, and India [22,42,43]. Interventions to address these factors include dengue awareness and community mobilization campaigns [44], water container covers, and programs to provide low-cost screening to homeowners. Paradoxically, we found that the use of mosquito nets was positively correlated with *Ae. aegypti* presence. We believe that this may be the result of a causal reverse in correlation, wherein bed nets are more likely to be used when mosquitoes are perceptibly present. Although *Aedes aegypti* have a small range and will bite during the day (in contrast to other mosquito genera such as *Anopheles*), suggesting that bed nets are an inappropriate intervention for dengue, another study on the mainland found that bed nets were protective against dengue infections [45]. 

We found that the use of air conditioning was positively associated with *Ae. aegypti* presence. This result is counterintuitive, because one would expect homes with air conditioning to have closed windows and subsequently fewer mosquitoes and lower dengue risk, as shown in prior studies [46]. It is possible that water buildup and puddles created by air conditioning units could create additional mosquito habitat, which would require further investigation. A more comprehensive understanding of air-conditioning practices would provide information on the context in which air-conditioning units are used versus installed. For example, if rooms are only cooled for a few hours a day, and, in the evening, open windows are used to cool houses, an air-conditioning unit would be a false signal of closed-window behavior. 

Water access and storage practices were among the most important household risk factors for both of our models for prior dengue infections and the presence of *Ae. aegypti*. This result concurs with previous work in mainland Ecuador [22,39]. Water access is a serious concern on the Galápagos Islands, which has a limited supply of fresh water [47,48]. As a result, many inhabitants store water around the home for daily use [49], creating the ideal habitat for *Ae. aegpyti* juveniles, especially in PB. A recent study on dengue transmission in Barbados, a water-scarce Caribbean island nation, found drought conditions increased the likelihood of dengue outbreaks [50]. The characteristics of containers positive for juvenile *Ae. aegypti* (uncovered water storage containers located outdoors) indicate that community clean-up campaigns focusing on the elimination of rubbish in the patio may have a limited effect on *Ae. aegypti* abundance, at least during the cool season, when this study was conducted. The creation of sealed (*Aedes*-proof) water storage containers used by households and located outdoors in the patio may be a more effective campaign than current rubbish removal efforts. This result highlights the importance of integrated household water management strategies in regions that are water-scarce and at risk of dengue.

We found differences in self-reported prior dengue infections, vector abundance, prevention strategies, sources of information, and risk perception between PA and PB. This suggests that there may be differences in a number of factors between the islands, including disease burden, community outreach programs, community awareness, and/or access to information between the two islands. The high proportion of homes with juvenile *Ae. aegypti* in PB indicates that there was significant risk of another dengue outbreak, even during the low-transmission season when this study was conducted. This increased risk is especially concerning due to the remote nature of San Cristobal, and the Galapagos Islands as a whole. 

It is likely that prior dengue infections reported from PB households were from the dengue outbreak that occurred in 2010, four years prior to this study, when most of the population was susceptible to dengue infections. The prevalence of past dengue infections self-reported by surveyed households (28%) was higher than the dengue prevalence reported by the Ministry of Health in 2010 (14.1%). The burden of disease during the outbreak was likely higher than reported by the Ministry of Health, since people rarely seek medical care for mild infections, resulting in underreporting by passive surveillance systems [1,51]. This discrepancy may also indicate that people are aware of the symptoms, but did not report those symptoms to authorities; thus, we see considerable self-diagnosed, but unreported dengue. A dengue surveillance study conducted in the same year in southern coastal Ecuador found that 32% of people with acute or recent dengue infections reported no dengue-like symptoms, and there were three additional dengue infections in the community for every case reported by the Ministry of Health [5]. This discrepancy is worth exploring in future work, as it indicates higher potential rates of infection than is informing current policies. 

One of the greatest public health challenges observed during this study was the implementation of uniform vector control and surveillance across the four populated islands, which are more than two hours apart by boat. The higher larval indices in PB may be due to less staffing and resources for vector control, since there were fewer total cases reported in PB than PA. Investigating the sources of these inconsistencies could provide insight into how to better mobilize and engage communities to promote the adoption of preventative behaviors across relatively isolated islands at risk from emerging infectious diseases.

The results of our study indicate that very few households are actively engaged in dengue control campaigns, despite the perceived importance and high awareness of the disease. A survey conducted in PA and PB in the same year found that nine out of ten people felt that they were not prepared for a future dengue outbreak, and about one-third of people reported that they were vulnerable to dengue infections (F. Ortega, personal communication). Studies from mainland Ecuador identified factors that influenced people’s willingness to engage in vector control, including social cohesion and leadership, the perceived role of the government versus the community, and the time and financial costs of household vector control [21,52]. Future studies that explore barriers to dengue control could be used to inform the development of community-based interventions to reduce the risk of dengue and other *Ae. aegypti* transmitted diseases, through strategies such as Communication for Behavioral Impact (COMBI) [44,53,54], which is supported by the Pan American Health Organization (PAHO) and the Ecuadorian government. 

The emergence of dengue in the Galápagos Islands has serious implications for the tourism-based economy of the islands. Tourism is an important and substantial source of income for inhabitants of the Galápagos Islands. The risk of disease could discourage tourists from visiting the islands. Furthermore, the high volume of tourists traveling from mainland Ecuador to the islands, particularly tourists passing through the city of Guayaquil, a dengue endemic city, could present a source of reintroduction of the virus to the islands.

Our findings highlight the importance of human movement in determining dengue transmission, particularly on islands where people travel regularly between islands. Individuals with frequent travel to other islands, as well as between the islands and the continent, may be considered to be at higher risk for dengue infection. Previous studies of dengue on islands emphasized the importance of preventing the transmission of dengue from the mainland [55], as well as reintroduction from surrounding islands [56]. Prior studies of West Nile virus risk in the Galápagos found that the transportation of infected mosquitoes via airplanes was the most likely means that the virus would be introduced to the islands [18]. The transmission dynamics between mainland and island populations may be exacerbated by seasonal differences in the epidemiology of travelers [57], which may support the restriction of travelers during significant outbreaks on the mainland. The recent emergence of chikungunya and Zika viruses highlights the importance of understanding these regional movement and transmission dynamics [58,59]. Our findings also suggest the importance of local, community-based movement dynamics in the transmission dynamics of dengue, as was explored in previous studies [23].

## 5. Conclusions

To our knowledge, this is the first study of dengue risk and *Ae. aegypti* presence in the Galápagos Islands. The findings that human movement within and between islands was important to reported dengue cases confirms concerns of this route of introduction and repeated transmission. Bolstering surveillance of tourism routes of entry would be useful to mitigate potential future introductions of the virus. We identified sources of dengue knowledge and perceptions of dengue risk among residents of the Galápagos Islands. We also assessed the importance of knowledge of prevention and identified a pervasive lack of community involvement in vector control campaigns. These are potential targets for policy and action that could be taken to reduce the risk of future dengue outbreaks. We found that, similar to studies conducted in mainland Ecuador, housing condition and water supply, access, and storage-related behaviors are important factors related to both self-reported dengue infections and *Ae. aegypti* presence. The water connection is particularly poignant for the Galápagos, where access to freshwater is a perpetual concern. Given the geographic challenges faced in distributed island vector management, this is a complicated and unique setting for dengue management. However, we identified similar targets for interventions as found in other studies, which could provide useful information for establishing combined outreach and direct intervention efforts in the public health arena. 

## Figures and Tables

**Figure 3:**
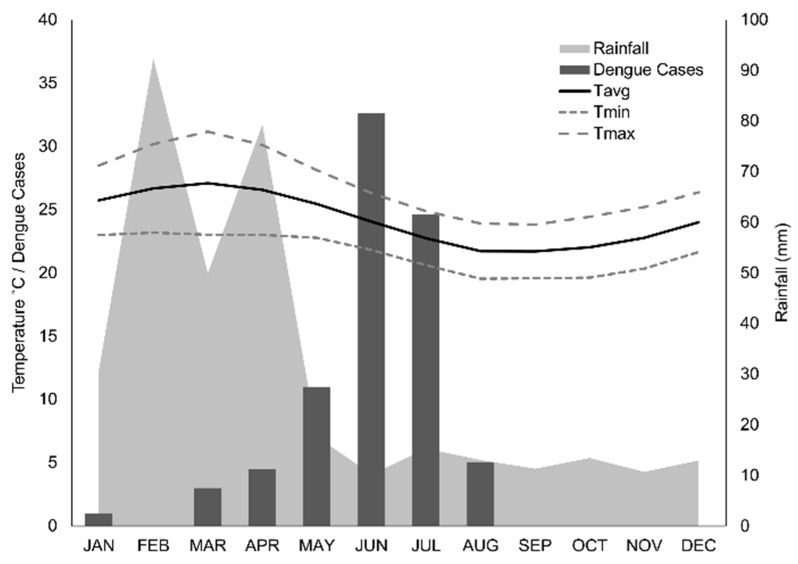
Seasonality of dengue transmission and climate in Puerto Ayora (2000–2012). We show average dengue cases reported by month, average monthly rainfall, and average monthly temperature (Tavg), with average minimum (Tmin) and maximum (Tmax) monthly temperature. Note that dengue cases appear to peak following the peak of temperature and rainfall, across the same 12-year time period.

**Table 1:** Socio-ecological parameters (mean and standard deviation) included in the multi-model selection framework to predict self-reported prior dengue infection and presence/absence of *Aedes aegypti* adults and/or juveniles at the household level for Puerto Ayora (PA) and Puerto Baquerizo Moreno (PB).

Parameter	PA		PB		*p*-Value
	*n*	Percent or Mean (±SD)	*n*	Percent or Mean (±SD)	
**Human Movement**					
Member of the household travels to the mainland ^1^	33	66%	39	78%	0.21
Member of the household travels between islands ^1^	15	30%	24	48%	0.09
Member of the household visits houses outside the neighborhood daily ^1^	9	18%	10	20%	1
**Demographics**					
Number of people per bedroom in household ^1,A^	41	2.07 (±1.37)	47	1.86 (±1.25)	0.45
Head of household age is less than 37 years ^1^	13	36%	7	18%	0.08
Head of household age is greater than 56 years ^1^	7	16%	11	37%	0.03
Female head of household ^1^	28	56%	12	24%	<0.01
Head of household is employed ^1^	36	72%	43	86%	0.03
More than 1 family sleeps on the property ^1^	25	53%	12	26%	0.01
Head of household earns more than the minimum wage ^1^	12	24%	14	28%	0.82
Head of household has a secondary level of education or higher ^1^	19	38%	19	38%	1
**Mosquito Abatement Practices**					
Covers water containers ^2^	31	62%	30	60%	1
Applies chemicals to standing water ^2^	5	10%	18	36%	<0.01
Eliminates standing water ^2^	2	4%	26	52%	<0.01
Disposes of trash on the property ^2^	6	12%	21	42%	<0.01
Fumigates house ^2^	9	18%	14	28%	0.34
Applies repellent ^2^	4	8%	13	26%	0.03
Closes windows and doors ^2^	0	0%	14	28%	<0.01
Cuts vegetation ^2,B^	0	0%	7	14%	0.01
Spreads burned diesel on floor/in puddles ^2,B^	0	0%	1	2%	1
Burns palosanto or insecticide coils ^2,B^	0	0%	2	4%	0.5
Uses bed nets	2	4%	27	54%	<0.01
**Housing Characteristics**					
Poorly maintained patio ^1,2^	22	44%	8	16%	<0.01
Poor overall condition of house (old, unpainted, uncared for) ^1,2,B^	2	4%	0	0%	0.5
Good overall condition of house (new, well-maintained) ^1,2^	19	38%	25	50%	0.31
Patio is shady (>50% shaded) ^1,2,B^	2	4%	6	12%	0.27
House is rented ^1,2^	12	24%	10	20%	0.81
Uses air conditioning to ventilate the household ^1,2^	6	12%	7	14%	1
No screens on windows and/or doors ^1,2^	9	18%	14	28%	0.34
Lives near abandoned homes or vacant lots ^1,2^	32	64%	44	88%	0.01
Household receives piped water from outside the home ^1,2^	11	22%	18	36%	0.19
There are daily or weekly interruptions in the water supply ^1,2^	22	44%	20	40%	0.84
Waste water disposal by a sewage system ^1,2,B^	1	2%	38	76%	<0.01
Stores water apart in containers other than in a cistern or elevated water tank (sometimes or always) ^1,2^	42	84%	28	56%	<0.01
**Knowledge and Attitudes**					
Aware of cases of dengue in the community ^1,2^	21	42%	27	54%	0.32
Considers dengue to be a severe disease ^1,2,B^	43	86%	49	98%	0.06
Knows that dengue is transmitted by a mosquito ^1,2^	47	94%	47	94%	1
Considers dengue to be a serious problem in the community ^1,2,B^	41	82%	47	94%	0.12
Perception that it is difficult to prevent dengue ^1,2^	6	12%	17	34%	0.02
Participated in dengue prevention campaigns ^1,2,B^	6	12%	5	10%	1
Source of dengue information: media (television, newspaper, radio) ^B^	46	92%	48	96%	0.67
Source of dengue information: Ministry of Health outreach (community health meetings, in clinics/hospitals, fliers)	28	56%	38	74%	0.09
Source of dengue information: social networks (family, friends) ^B^	6	12%	7	14%	1

The *p*-values ≤ 0.05 indicate significant differences between the PA and PB study areas; values were calculated using Pearson’s chi-square test with Yates continuity correction, unless otherwise indicated. Model input variables for each model indicated with subscripts (^1^ = self-reported dengue, ^2^ = *Aedes aegypti* presence). ^A^ The *p*-values were calculated by Student’s independent *t*-test; ^B^ the *p*-values were calculated by Fisher’s exact test.

**Table 2:** *Aedes aegypti* (AA) indices in August–September 2014 for households in Puerto Ayora (PA) and Puerto Baquerizo Moreno (PB).

Vector Indices	Total	PA		PB		*p*-Value
	*n*	*n*	%	*n*	%	
Houses inspected	100	50		50		
Total containers with water	248	119		129		
Houses with adult AA	9	2	4.00%	7	14.00%	0.16
Houses with containers with juvenile AA	13	3	6.00%	10	20.00%	0.012 *
Total containers with juvenile AA	16	3	2.50%	13	10.10%	0.019 *
Breteau index		6		26		
House index		6		20		

* A *p*-value ≤ 0.05 indicates a significant difference between the study areas; values were calculated using Fisher’s exact test. Breteau index represents the number of containers found with juvenile AA per 100 homes. House index represents the number of homes found to have juvenile AA per 100 homes.

**Table 3:** Summary of the best-fit model * for self-reported prior dengue infections.

Factor	Estimate	SE	*p*-Value	OR (95% CI)	VIF
Intercept	−7.02	1.98	<0.001		
People per bedroom	1.28	0.39	<0.001	3.60 (0.00–8.80)	2.11
Head of household earns more than minimum wage	3.23	1.09	<0.01	25.31 (0.00–301.64)	2.08
Visit other neighborhoods daily	−2.95	1.37	0.031	0.05 (0.00–0.53)	1.49
Travel to island	3.16	1.06	<0.01	23.57 (0.00–268.08)	2.17
Daily or weekly interruption in the piped water supply	2.18	1.00	0.029	8.85 (0.00–84.26)	1.96
Aware of cases of dengue in the neighborhood	2.54	1.16	0.028	11.85 (0.00–107.67)	1.69
Considers dengue to be a serious problem in the community	−2.90	1.42	0.041	0.06 (0.00–0.75)	1.74
Waste water disposal by a sewage system	1.47	0.87	0.092	4.35 (0.00–29.21)	1.47

* Akaike’s information criterion (AIC) corrected for small sample size (AICc) = 70.04, κ = 12.52; SE: standard error; OR: odd ratio; CI: confidence interval; VIF: variance inflation factor.

**Table 4:** Summary of the top model * for the presence of adult and juvenile *Aedes aegypti*.

Factor	Estimate	SE	*p*-Value	OR (95% CI)	VIF
Intercept	−22.81	2815.28	0.994	-	
Uses mosquito nets	3.25	1.20	0.007	25.90 (2.47–271.24)	2.07
Uses repellant	−2.37	1.52	0.118	0.09 (0.01–1.83)	1.67
Covers water containers	−3.39	1.24	0.006	0.034 (0.003–0.38)	2.19
Closes windows and doors	−3.10	2.02	0.126	0.05 (0.001–2.38)	1.85
Uses chemicals to treat water	−2.89	1.52	0.057	0.06 (0.003–1.10)	2.02
Perception that dengue is a severe disease	19.61	2815.28	0.994	-	1
Air conditioning	5.27	1.78	0.003	194.06 (5.98–6293.60)	3.87
Water is piped into and out of the house	2.47	1.28	0.055	11.80 (0.95–146.11)	2.27
Dengue is difficult to prevent	2.26	0.97	0.020	9.61 (1.42–64.95)	1.35

* AICc = 60.14, κ = 8.68.

## Supplementary Materials

The following are available online at https://www.mdpi.com/1660-4601/16/5/682/s1: Table S1: Competing top models for predictions of self-reported dengue (Prior Dengue); Table S2: Competing top models for predictions of *Aedes aegypti* presence (AA).
