# Supplementary material for: Socio-Ecological Factors Associated with Dengue Risk and Aedes aegypti Presence in the Galápagos Islands, Ecuador
Source: Int J Environ Res Public Health. 2019 Feb 26;16(5):682. doi: 10.3390/ijerph16050682 (PMC6427784; doi:10.3390/ijerph16050682)
Supplement: Supplementary file 1 [file ijerph-16-00682-s001.pdf]

Table 1. Competing top models for predictions of self-reported dengue (Prior Dengue).

	**Model**	**AICc**	**Weights**	**Δ AICc**
1	PriorDengue ~ 1 + pplrm + awarecases + visitbarriodaily + travisl + pipedinterrupt + problema + sewer + moreminwage	70.04	0.07	0
2	PriorDengue ~ 1 + pplrm + awarecases + visitbarriodaily + travisl + pipedinterrupt + problema + noscreens + sewer + moreminwage	70.35	0.06	0.31
3	PriorDengue ~ 1 + pplrm + awarecases + visitbarriodaily + travisl + pipedinterrupt + problema + sewer + gt1hh + moreminwage	70.44	0.06	0.40
4	PriorDengue ~ 1 + pplrm + awarecases + visitbarriodaily + travisl + pipedinterrupt + problema + moreminwage	70.75	0.05	0.71
5	PriorDengue ~ 1 + pplrm + awarecases + visitbarriodaily + storewater + travisl + pipedinterrupt + problema + sewer + moreminwage	70.98	0.05	0.95
6	PriorDengue ~ 1 + pplrm + awarecases + patioshade + visitbarriodaily + travisl + pipedinterrupt + problema + sewer + moreminwage	71.13	0.04	1.10
7	PriorDengue ~ 1 + pplrm + goodhouse + awarecases + visitbarriodaily + travisl + pipedinterrupt + problema + sewer + moreminwage	71.15	0.04	1.11
8	PriorDengue ~ 1 + pplrm + awarecases + severe + visitbarriodaily + travisl + pipedinterrupt + problema + sewer + moreminwage	71.19	0.04	1.15
9	PriorDengue ~ 1 + pplrm + badpatio + awarecases + visitbarriodaily + travisl + pipedinterrupt + problema + sewer + moreminwage	71.40	0.04	1.35
10	PriorDengue ~ 1 + pplrm + goodhouse + awarecases + visitbarriodaily + travisl + pipedinterrupt + problema + moreminwage	71.50	0.04	1.46
11	PriorDengue ~ 1 + pplrm + awarecases + visitbarriodaily + travisl + pipedinterrupt + problema + sewer + oldhh + moreminwage	71.73	0.03	1.69
12	PriorDengue ~ 1 + pplrm + goodhouse + awarecases + visitbarriodaily + travisl + pipedinterrupt + problema + moreminwage + secondedu	71.76	0.03	1.72
13	PriorDengue ~ 1 + pplrm + awarecases + visitbarriodaily + travcont + travisl + pipedinterrupt + problema + sewer + moreminwage	71.76	0.03	1.72
14	PriorDengue ~ 1 + pplrm + goodhouse + awarecases + visitbarriodaily + travisl + pipedinterrupt + problema + sewer + moreminwage + secondedu	71.89	0.03	1.85
15	PriorDengue ~ 1 + pplrm + awarecases + visitbarriodaily + AC + travisl + pipedinterrupt + problema + sewer + moreminwage	71.96	0.03	1.93

Table S2. Competing top models for predictions of *Aedes aegypti* presence (AA).

	Model	AICc	Weights	ΔAICc
1	AA ~ 1 + mallas + repelente + tapar + cerrar + quimicos + severe + prevdifficult + AC + pipedout	60.14	0.071	0
2	AAt ~ 1 + mallas + repelente + tapar + cerrar + quimicos + severe + prevdifficult + patioshade + AC + pipedout	60.78	0.05	0.64
3	AA~ 1 + mallas + repelente + tapar + quimicos + severe + prevdifficult + AC + pipedout	60.82	0.05	0.67
4	AA ~ 1 + mallas + tapar + cerrar + quimicos + severe + prevdifficult + AC + pipedout	60.82	0.05	0.67
5	AA ~ 1 + mallas + repelente + tapar + cerrar + quimicos + eliminar.agua + severe + prevdifficult + AC + pipedout	61.52	0.04	1.38
6	AA ~ 1 + mallas + repelente + tapar + cerrar + quimicos + badpatio + severe + prevdifficult + AC + pipedout	61.74	0.03	1.59
7	AA ~ 1 + mallas + repelente + tapar + cerrar + quimicos + goodhouse + severe + prevdifficult + AC + pipedout	61.76	0.03	1.61
8	AA~ 1 + mallas + repelente + tapar + cerrar + quimicos + badhouse + severe + prevdifficult + AC + pipedout	61.95	0.03	1.81
9	AA ~ 1 + mallas + repelente + tapar + cerrar + quimicos + severe + prevdifficult + AC	62.12	0.03	1.98
